# Persistence and Diffusion of *mec*C-Positive CC130 MRSA Isolates in Dairy Farms in Meurthe-et-Moselle County (France)

**DOI:** 10.3389/fmicb.2019.00047

**Published:** 2019-01-30

**Authors:** Jacques Bietrix, Camille Kolenda, Anaïs Sapin, Marisa Haenni, Jean-Yves Madec, Michèle Bes, Céline Dupieux, Jason Tasse, Fréderic Laurent

**Affiliations:** ^1^Cliniques Vétérinaires de l’Arche, Valence, France; ^2^Pathogénie des Infections à Staphylocoque, Centre International de Recherche en Infectiologie, Lyon, France; ^3^Institut des Agents Infectieux, Laboratoire de Bactériologie, Hospices Civils de Lyon, Lyon, France; ^4^Agence Nationale de Sécurité Sanitaire de l’Alimentation, de l’Environnement et du Travail – Université de Lyon, Lyon, France; ^5^Centre National de Référence des Staphylocoques, Hospices Civils de Lyon, Lyon, France; ^6^Institut des Sciences Pharmaceutiques et Biologiques, Lyon, France

**Keywords:** *Staphylococcus aureus*, *mec*C, dairy cow, methicillin resistance, milk, carriage

## Abstract

**Background:** Methicillin resistance in *Staphylococcus aureus* (MRSA) is classically conferred by the acquisition of the *mec*A gene encoding an additional penicillin binding protein with low affinity for beta-lactams. A *mec*A variant, named *mec*C, was described in 2011. MRSA isolates harboring *mec*C of both animal and human origin have since been collected in different European countries. In France, animal cases were reported in 4 dairy farms between 2008 and 2013 in the Meurthe-et-Moselle county, all located in a 30 km perimeter, suggesting a possible dissemination of *mec*C-positive MRSA strains. We performed a prospective study to evaluate the local epidemiology of such strains in terms of (i) dissemination among animals, humans and in the environment, and (ii) persistence in Meurthe-et-Moselle dairy cattle farms.

**Methods:** The 4 French dairy farms with previous reports of *mec*C-positive MRSA strains and 14 farms in the same perimeter were included in this study. In each farm, nasal swabs, rectal swabs and milk samples were collected from 10 randomly selected cows, as well as nasal samples from family pets, volunteer farmers and veterinarians. One farm (E0), in which *mec*C-MRSA isolates were detected, was selected to study more deeply the dissemination of *mec*C-positive strains within the farm. After pre-enrichment of swabs and milk, they were subcultured on MSSA/MRSA chromogenic selective agar plates. *S. aureus* colonies were tested with a multiplex PCR to detect the *mec*A and *mec*C genes. The *mec*C-positive strains were characterized using DNA microarray.

**Results:** *mec*C-positive strains were recovered in four farms, corresponding to the ones with previous reports of *mec*C-positive MRSA strains, and originated only from dairy cow samples. The screening in the E0 farm showed that 22% of the dairy cows carried *mec*C-positive MRSA. Three strains were also isolated from the environmental samples. All *mec*C-positive strains belonged to the clonal complex CC130 and harbored the same spa-type t1736.

**Conclusion:** This study found that *mec*C-positive MRSA isolates are able to persist within the same farms for several years after being introduced in this setting and are able to widely disseminate but only among dairy cows suggesting that milking machines might be a key player.

## Introduction

*Staphylococcus aureus* is a Gram-positive bacteria commonly found in the commensal flora of humans and many animal species. The prevalence of asymptomatic nasal carriage in adults is approximately 30% (Kluytmans et al., 1997; Sivaraman et al., 2009). *Staphylococcus aureus* is also an opportunistic pathogen responsible for infections ranging from suppurative skin infections to life-threatening septicemia, pneumonia, and osteomyelitis. In animals, *S. aureus* infections are rare, except for dairy cows for which it represents a major cause of chronic mastitis inducing major economic loss (Espinosa-Gongora et al., 2012; Pantosti, 2012). Beta-lactams are used as first-line treatment to treat these infections in both humans and animals. As a consequence, methicillin resistant *Staphylococcus aureus* (MRSA*)* has emerged and spread widely since 1961. This resistance is classically conferred by the acquisition of a genetic mobile element, called SCC*mec,* containing the *mec*A gene encoding an additional penicillin binding protein (PBP2a) which presents a reduced affinity for all beta lactams (International Working Group on the Classification of Staphylococcal Cassette Chromosome Elements [IWG-SCC], 2009). In 2011, a divergent *mec*A homolog within a novel SCC*mec* type XI element, originally named *mec*A_LGA251_ and subsequently designated as *mec*C, was described in bovine and human isolates from the United Kingdom and Denmark (García-Álvarez et al., 2011).

Methicillin resistance in *Staphylococcus aureus* isolates harboring *mec*C of both human and animal origin have since been collected in different European countries (Paterson et al., 2014a; Aires-de-Sousa, 2017). In humans, most of the retrospective studies found a low but variable prevalence of *mec*C-positive MRSA (Cuny et al., 2011; Basset et al., 2013; Paterson et al., 2014b). However, in Denmark, where all human MRSA strains are collected by the national reference laboratory, a marked increase of human *mec*C-MRSA strains was observed between 2003 and 2011 (Moller et al., 2017). Furthermore, in this country, zoonotic transmission of *mec*C-MRSA from livestock to humans was demonstrated by epidemiological follow-up and whole genome sequencing (Harrison et al., 2013; Petersen et al., 2013).

Dairy cows are a reservoir and source of transmission to humans of *mec*C-positive MRSA strains. Although MRSA is rarely involved in bovine mastitis, some published data suggest that the proportion of *mec*C-positive isolates among these scarce MRSA strains is unexpectedly high in France. In a study gathering 1549 coagulase-positive staphylococci strains between 2011 and 2013, 10 MRSA strains were isolated including 4 that were *mec*C-positive. These four isolates belonged to the same clone and originated from dairy farms all situated within a 30 km perimeter (Meurthe-et-Moselle county) around the city of Nancy (North-East France) (Haenni et al., 2014). Based on these results, we performed a prospective study to evaluate the local epidemiology of such strains in terms of (i) dissemination among animals, humans and in the environment, and (ii) persistence in Meurthe-et-Moselle dairy cattle farms.

## Materials and Methods

### Samples and Processing

Four dairy farms with a previous report of *mecC*-positive MRSA strains were included (Laurent et al., 2012; Haenni et al., 2014). In addition, 14 other farms in the same geographic perimeter were screened. First, milk, nasal and rectal samples were collected from 10 randomly selected cows in each farm. Nasal samples were also collected from volunteer farmers and veterinarians in 14 farms, and family pets in 10 farms. Secondly, one of the farms showing samples containing *mec*C-positive MRSA strains, designated E0, was selected to investigate more deeply animal, human, and environmental spread of such strains in the herd. In this farm, all the cows (45 dairy cows and 30 breeding cows), calves (*n* = 3), dogs (*n* = 3), other family pets (*n* = 21), the farmer and their family were sampled as previously described. Twenty environmental samples were also collected from the farm and farmer’s house. Sterile swabs containing Amies liquid medium (Venturi Transystem^®^, Copan, Brescia, Italy) were used for nasal (both nostrils) and rectal sampling. Samples were stored at +4°C for up to 7 days before testing. They were discharged in 2 mL of Brain-Heart infusion (BHI, Becton Dickinson, Le Pont de Claix, France) with 5% NaCl and incubated for 24 h at 37°C. Milk samples were collected aseptically by manual milking and stored at +4°C for up to 24 h. They were diluted twofold in BHI with 10% NaCl and incubated for 24 h at 37°C. Environmental samples were collected by rubbing sterile wipes on several surfaces for 1 min (sterile cloth impregnated with Ringer’s solution, Sodibox, Névez, France). The wipes were incubated in 100 mL of BHI with 5% NaCl for 24 h at 37°C.

In the E0 farm, the milk of the cows carrying *mec*C-positive MRSA was again sampled after 1 month in order to assess the clinical impact of this colonization (30 mL per udder quarter) by performing bacteria quantification and determination of the cellularity (Fossomatic^®^FC, Nanterre, France). A quarter was considered to be sub-clinically affected when clinical signs were not present and the number of cells in the milk was greater than the threshold value of 300 000 cells/mL.

For all the samples performed in this study, informed consent was obtained from the owners of cows and pets.

### Identification and Characterization of *mec*C-Positive MRSA Strains

After incubation, 10 μL of broth were plated onto two selective chromogenic media, namely Brilliance^®^Staph 24 agar (Oxoid, Dardilly, France) and ChromID^®^MRSA agar (BioMérieux, Marcy l’Etoile, France) for MSSA and MRSA screening, respectively. The plates were incubated overnight at 37°C. Suspected *S. aureus* colonies were isolated and identified using MALDI-TOF (Vitek^®^MS, BioMérieux). A multiplex PCR was performed on each *S. aureus* isolate to detect the *mec*A and *mec*C genes (Stegger et al., 2012). Then, MRSA isolates were typed using *spa*-typing as described previously (Harmsen et al., 2003). The x region of the *spa* gene was amplified by PCR followed by sequencing. *spa* types were determined with the Ridom Staph Type software (Ridom GmbH, Würzburg, Germany), which automatically detects *spa* repeats and assigns a *spa* type. Their genetic backgrounds, their *agr* type and the presence of genes coding for virulence and resistance factors were determined using DNA microarray (Staphytype^®^, Alere Technologies GmbH, Jena, Germany). The hybridization pattern was analyzed with Arraymate^®^(Alere Technologies GmbH).

### Antimicrobial Susceptibility Testing

Resistance to oxacillin, cefoxitin, moxalactam, erythromycin, fosfomycin, gentamicin, kanamycin, lincomycin, linezolid, ofloxacin, penicillin, pristinamycin, rifampin, sulfamethoxazol, teicoplanin, tobramycin, and vancomycin was assessed using disc diffusion on Mueller-Hinton 2 agar (Bio-Rad, Marnes-la-Coquette, France) according to the guidelines of the Antibiogram Committee of the French Society for Microbiology (CA-SFM guidelines, 2018).

## Results

### Prevalence of MRSA in Meurthe-et-Moselle Dairy Farms

*Staphylococcus aureus* isolates were collected in 12 farms (MSSA in 8 farms, MRSA in 4 farms). No *mec*A-positive MRSA isolates were found. The *mec*C-positive MRSA prevalence among the 18 farms was 22.2% (4/18 farms). Of note, these four farms corresponded to the ones in which *mec*C-positive MRSA isolates were reported between 2008 and 2013 (Laurent et al., 2012; Haenni et al., 2014). All MRSA strains were isolated from cow samples and MRSA carriage was never observed in farmers, family pets, or veterinarians. In the four farms with *mec*C-positive MRSA a variable number of the 10 sampled cows carried *mec*C-positive MRSA (1/10, 2/10, 4/10, and 6/10). MRSA was isolated in the milk for 10 cows, nasal swab for 1 cow, rectal swab for 1 cow, and in both milk and nose for 1 cow (considered as only one strain in this study) (Table 1).

**Table 1:** Description of the origin of the *mec*C-positive MRSA strains recovered in this study.

Farm	N° of cows carrying *mec*C-positive MRSA (*x*/10)	N° of positive milk samples (*x*/10)	N° of positive rectal swab samples (*x*/10)	N° of positive nasal swab samples (*x*/10)
3	2	1		2
13	4	3	1	
17^∗^	6	6		
18	1	1		
Total	13	11	1	2


*^∗^This farm corresponds to the farm E0*.

### Diffusion of *mec*C-Positive MRSA in a Dairy Farm (E0) and Clinical Impact on the Development of Subclinical Mastitis

The *mec*C-positive MRSA prevalence among dairy cows was 22% (10/45). Isolates were collected only from milk. None of the breeding cows, calves, dogs, or humans were found to be positive. Three strains were isolated from the environmental samples (the milking room gate, bulk tank milk, and coffee pot handle samples).

Bacterial colonization was confirmed after 1 month in the udder for 7/10 dairy cows carrying *mec*C-positive MRSA; *mec*C-positive MRSA was detected in 16 udder quarters including 13 quarters with a subclinical mastitis (more than 300 000 cells/mL in the milk). The correlation between the cellularity and the number of MRSA colonies per mL was significant (Spearman correlation test, *p* < 0.0001).

### Antibiotic Susceptibility Profile and Characterization of Bovine *mec*C-Positive MRSA Strains

In total, 26 *mec*C-positive MRSA strains were isolated. They all belonged to the clonal complex CC130 and *spa*-type t1736. All strains had a type 3 *agr* allele and type XI SCC*mec*. Methicillin resistance was detected for 88,5% (23/26) of the strains using a cefoxitin disc. All strains were susceptible to the other antibiotics tested.

The DNA microarray hybridization profile confirmed the presence of the *mec*C gene and absence of *mec*A gene for the 26 strains tested. The virulence profile was identical for all isolates. Genes coding for hemolysins (*hla*, *hlb*, and *hld*), leucocidins (*luk*F, *luk*S, *luk*D, *luk*Y, and *hlg*A), immune evasion proteases (*aur*, *spl*A, *spl*B, and *spl*E), binding proteins (*bbp*, *clf*A, *clf*B, *ebh*, *fnb*A, and *fnb*B), exfoliative toxin (*edin*B), biofilm and capsule proteins (*cap*8, *ica*-operon) were detected. Absence of PVL, superantigens, and human specific immune evasion cluster (IEC) coding genes was found for all isolates.

## Discussion

We report herein the first prospective prevalence study of *mec*C-positive MRSA in French dairy farms. Although the prevalence appears to be low in some European countries (between 0.01 and 0.8% of bovine *S. aureus* strains in Finland and Sweden) (Gindonis et al., 2013; Unnerstad et al., 2013), a large study performed in 465 dairy farms in England and Wales found a prevalence of 2.15% (Paterson et al., 2014c). In the present study, we detected *mec*C-positive MRSA in 22.2% of the farms. However, we screened four farms which were already known as colonized by *mec*C-positive MRSA in the past. This introduced a bias in the prevalence evaluation, and the results must be carefully interpreted considering the limited number of farms included and the specific area targeted in this study. Nevertheless, this high rate in a narrow geographical area is worrisome and suggests a widespread diffusion of *mec*C-positive MRSA in the Meurthe-et-Moselle county and calls for a larger epidemiological investigation to ascertain the true prevalence in France. The occurrence of *mec*C-positive MRSA in a very small geographic area gives credit to a possible clustering of *mec*C-positive MRSA cases in some specific herds. Nevertheless, *mec*C-positive MRSA were not detected in the 14 other farms, very close to the 4 farms colonized with this kind of isolates suggesting that, if the intra-farm dissemination is important, the inter-farm spreading is limited. All the isolates of this study belonged to CC130, a bovine-specific clonal complex which is the most prevalent CC among *mec*C-positive MRSA (Paterson et al., 2014a), and to *spa*-type t1736, reported in previous cases of bovine mastitis in this county and in the same farms several years ago (Haenni et al., 2014). These data suggest that *mec*C-positive MRSA isolates are able to persist within the same farms for a long time after being introduced in this setting.

The evaluation of the diffusion of *mec*C-positive MRSA strains within a dairy farm found that the carriage rate among dairy cows of the E0 farm was 22%, which is consistent with a previous study that reported that 28% of 56 cows were colonized in a Bavarian dairy farm (Schlotter et al., 2014). Herein, MRSA strains were mostly isolated from milk samples (11 cows among the 13 colonized by MRSA) suggesting that the transmission within the farm could occur during milking and is likely to be facilitated by the use of milking machines. This hypothesis is supported by the finding that *mec*C-positive MRSA carriage was not observed in other animals of the same farm, i.e., breeding cows, calves, dogs. Moreover, the presence of such strains in environmental samples of this farm shows that persistence on surfaces and indirect transmission with milking material is possible. Only one Spanish study previously described *mec*C-positive MRSA strains in the environment and collected isolates from river water (Concepción Porrero et al., 2014). It could be interesting to further explore the persistence capacity of these strains in the environment and their fitness to estimate the risk of dissemination by indirect transmission.

No *mec*C-positive MRSA strains were detected in human samples (farmers and their family, veterinarians) suggesting that, even if the prevalence of *mecC*-positive MRSA is high in cows, the incidence of transmission to humans remains low. These results corroborate those of a previous study that found that no *mec*C-positive MRSA carriers were identified among a large population of cattle veterinarians in which the prevalence of *mec*A-positive MRSA nasal carriage was 2.6% (Paterson et al., 2013). Nevertheless, several cases of zoonotic transmission to humans of ovine and bovine CC130 MRSA strains were reported, highlighting the capacity of this CC to infect humans, and then representing a potential professional risk for dairy farmers (Harrison et al., 2013; Paterson et al., 2013). The lack of the genes *sak*, *chp*, and *scn*, which are involved in the human-specific IEC and are considered as an indicator of an adaptation of animal *S. aureus* strains to humans (Sung et al., 2008), could explain the surprisingly low rate of human colonization by *mec*C-positive MRSA in the present study. However, all the strains harbored an untruncated *hld* gene which is able to receive the prophage containing the *sak*, *chp*, and *scn* genes, suggesting a possible adaptation of these strains and then a potential risk of transmission to humans and spread, as for the re-adaptation to human of the swine ST398 MRSA clone (Price et al., 2012). As previously described, *mec*C-positive MRSA strains in the present study did not produce any major human virulence factor such as PVL or superantigens (Cuny et al., 2011; Sabat et al., 2012).

Methicillin resistance was not detected for all strains in this study using disk diffusion. Several studies previously reported that *mec*C-MRSA strains could be misclassified as MSSA by conventional AST methods (Cartwright et al., 2013; Skov et al., 2014; Kolenda et al., 2017). The discrepancy between the presence of the *mec*C gene and the low level of beta-lactam resistance has been related to the high transcription repression of the *mec*C gene by the *mec*I/*mec*R system described in *mec*C-positive MRSA strains (Kim et al., 2012). Kim et al. (2012) also demonstrated that the additional penicillin binding protein encoded by *mec*C (PBP2c) was less stable than the PBP2a encoded by *mec*A at 37°C, leading to a loss of sensitivity for the detection of methicillin resistance. Taken together, these data suggest that *mec*C-positive MRSA strains may be under-detected in humans and animals.

To conclude, persistence and diffusion of CC130 *mec*C-positive MRSA seem to be unexpectedly important in affected dairy farms inducing subclinical mastitis, economic burden, and professional risk for breeders even if no human isolates were recovered in our study. If human carriage of such strains seems very low up to now, the acquisition of human-specific virulence factors such as *sak*/*chp*/*scn* genes in the future could potentially promote their dissemination. An alternative scenario could be the transfer of SCCmec type XI cassette from an animal clone, such as CC130, to a human-adapted clone.

## Ethics Statement

This national veterinary epidemiological study performed in France did not require any ethical approval. Samples were performed by veterinarians in farms based on owner’s volunteering. They consisted in non-invasive samples (rectal and nasal swabbing, milk collection) performed on animals that were not raised for the purpose of this study.

## Author Contributions

JB, FL, and MH designed the study. JB and JT collected the samples. JB, CK, AS, JT, MB, and CD contributed to microbiology analysis. JB, CK, JT, and FL analyzed the results. JB, CK, and JT prepared the manuscript. All the authors revised and approved the manuscript.

## Conflict of Interest Statement

The authors declare that the research was conducted in the absence of any commercial or financial relationships that could be construed as a potential conflict of interest.
